# Clinical management of severe molar–incisor hypomineralisation using CAD/CAM fabricated PMMA crowns: a case report

**DOI:** 10.2340/biid.v13.45566

**Published:** 2026-03-12

**Authors:** Vignesh Ravi, Madhur Sharma

**Affiliations:** a Private Dental Practice, Chennai, India; b ESIC Medical College and Hospital, Alwar, Rajasthan, India

**Keywords:** MIH, PMMA crowns, CAD/CAM, paediatric crown

## Abstract

**Background:**

Molar-incisor hypomineralisation (MIH) is a well-documented developmental enamel disorder, typically involving first permanent molars (FPMs) and incisors. However, atypical presentations with extensive involvement of other permanent teeth remain uncommon and poorly understood leading to management challenges.

**Materials and methods:**

This case report describes a severe presentation of MIH in a 10-year-old male, with a significant medical history of high-grade fever and febrile seizures during infancy. The patient reported normal timing of permanent tooth eruption, followed by progressive post-eruptive breakdown, affecting all permanent teeth except the premolars and second permanent molars. Clinical and radiographic evaluations confirmed severe MIH with substantial loss of enamel structure, especially in FPMs and incisors, which led to severe aesthetic and functional concerns. A comprehensive treatment plan was devised. CAD/CAM technology was employed to fabricate and deliver polymethyl methacrylate (PMMA)-based crowns for the affected teeth to restore aesthetics and function.

**Discussion:**

The patient was monitored over 24 months, demonstrating satisfactory outcomes of PMMA crowns in terms of durability, mastication, appearance and psychological benefit, indicating a successful restorative outcome. This case highlights the severe presentation of MIH, possibly linked to early childhood systemic illnesses, and individualised management of MIH, particularly in young paediatric patients.

KEY MESSAGES

1. Minimally invasive CAD/CAM PMMA crowns offer effective functional and aesthetic rehabilitation in atypical severe MIH.
2. Early personalised management of MIH enhances both oral health and psychosocial well-being in children.

## Introduction

Developmental defects of enamel (DDE) are among the most frequently reported developmental anomalies affecting both primary and permanent teeth. Evidence from systematic reviews and meta-analyses shows a wide global prevalence range, reported at 3.9–81.5% in primary dentition and 9–63% in permanent dentition [1, 2]. Among these defects, molar-incisor hypomineralisation (MIH) has become one of the most widely recognised and clinically challenging subtypes [1]. According to Weerheijm et al., MIH is defined as a hypomineralisation of systemic origin of one to four permanent first molars frequently associated with affected incisors [3]. MIH’s reported prevalence ranges greatly, from 3% to 40% [4]. However, according to current meta-analyses, between 13% and 14% of children worldwide suffer from MIH [5, 6]. The likelihood of MIH impacting permanent incisors rises with the number of affected first permanent molars (FPMs) [7, 8]. Studies have demonstrated that the tips of permanent cuspids may exhibit defects similar to MIH, as cuspid mineralisation occurs during the same developmental period as FPMs and incisors; however, this suggestion remains theoretical and has not yet been substantiated by evidence [9, 10].

Premature birth, extremely low birth weight, fever, pregnancy complications, genetics and the use of antibiotics are some of the reasons linked to MIH [4, 11]. There is still much discussion and research surrounding the exact aetiology of MIH including whether a potential association exists between MIH and dietary vitamin D deficiency [4, 11, 12]. Clinically, MIH enamel defects may vary from creamy-white to yellow-brown opacities, and they always exhibit a clear distinction between the affected and healthy enamel. In MIH, the enamel has normal thickness, but it is structurally fragile, porous, and brittle, leading to loss of tooth structure under masticatory stresses [3, 13, 14]. This compromised integrity of the enamel can lead to tooth hypersensitivity, often causing children to avoid toothbrushing and other dental care routines [14]. Because of this poor oral hygiene, the teeth are more vulnerable to the development of caries at a younger age [15–17].

The prognosis of teeth with MIH varies greatly depending upon tooth type and clinical presentation. FPMs with yellow-brown opacities, post-eruptive breakdown and hypersensitivity convey a poor prognosis for long-term survival without intervention [11], hence, early preventive care and restorative treatments are crucial for maintaining these teeth and enhancing the patient’s quality of life [18]. Preventive measures include the use of self-applied and professionally applied topical fluoride that can aid in strengthening of the tooth [19, 20]. Restorations, crowns and extraction are the treatment options available for severe MIH [11]. Despite the availability of these restorative possibilities, it is clear that children with MIH often receive more treatment than children without MIH. Hence, regular dental check-ups are essential for prompt intervention [21].

Recent advancements in digital dentistry have led to the increased use of CAD/CAM technology and 3D printing for full-coverage restorations, including PMMA crowns, which are now considered a viable option for long-term temporary or interim restorations in young MIH patients [22]. These techniques enable precise fabrication with reliable occlusal and proximal contact, improved marginal fit, reduced chair-time, predictable aesthetics, and minimal tooth preparation [23, 24]. Despite these advantages and growing accessibility, clinical evidence on the long-term performance of CAD/CAM PMMA crowns remains limited.

This case report describes the management of severe MIH involving all FPMs, incisors, and canines in a paediatric patient with a history of vitamin-D–deficiency–related hypocalcaemia. A novel full-coverage restorative approach was adopted to restore function and aesthetics, with successful results maintained over 24 months.

## Case presentation

This manuscript has followed the CARE checklist case reporting guidelines [25]. The study was conducted in accordance with the ethical principles outlined in the Declaration of Helsinki and the patient and caregivers provided appropriate patient consent forms.

A 10 year‑old male patient presented with a chief complaint of hypersensitivity to cold food and normal air while talking or travelling in his upper front teeth for the past 1.5 years. The patient had no apparent issues until the eruption of his FPMs and incisors. However, shortly after these teeth emerged, they began to chip away in small fragments, developing a yellowish-brown discolouration that affected both anterior and posterior teeth, leading to hypersensitivity.

The child’s mother reported no abnormalities during the gestation or birth. The child was born in normal health conditions, but review of his medical history revealed that during his late first year of life, he experienced high-grade fever, frequent febrile seizures and was diagnosed with severe hypocalcaemia secondary to vitamin D deficiency (as per the medical record). He was subsequently placed on vitamin D supplementation and anticonvulsant therapy. The child’s mother also reported that the child experienced pain during toothbrushing—particularly in the affected molars and incisors—due to sensitivity to the bristles, resulting in poor oral hygiene and persistent bad breath.

**Figure 1:**
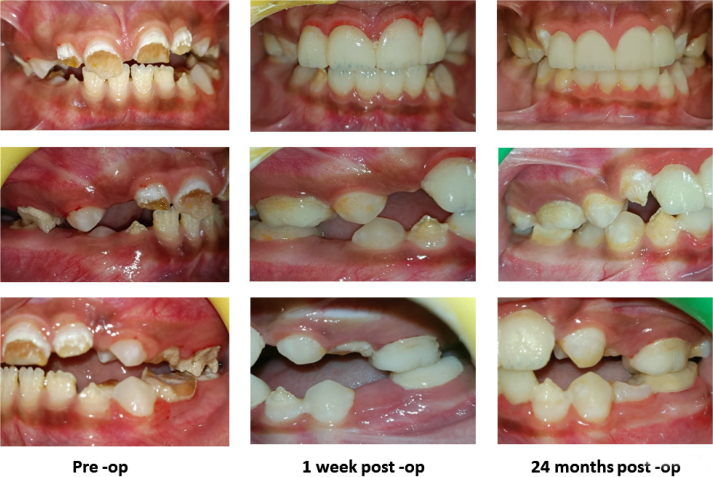
Intraoral photograph of MIH-affected teeth. Pre-op; 1 week Post-op; 24 months Post-op.

**Figure 2:**
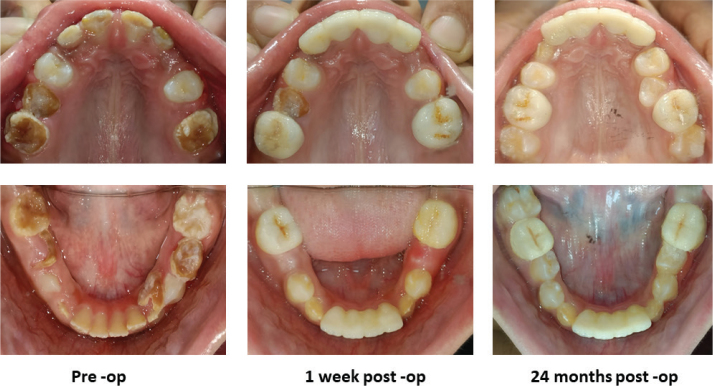
Intraoral occlusal photograph of maxilla and mandible showing MIH-affected teeth. Pre-op; 1 week Post-op; 24 months Post-op.

**Figure 3:**
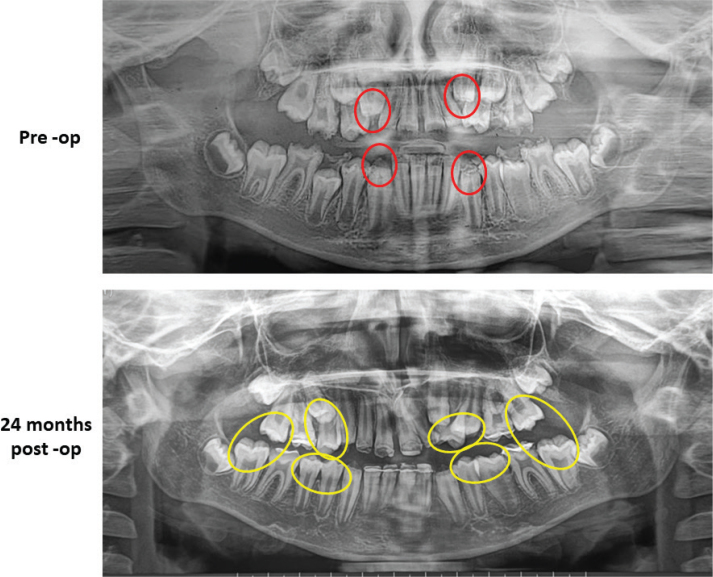
Pre-op; 24 months Post-op. Red circle- shows the defective, combined hypoplastic- hypomineralised incisal edge of the canines. Yellow circle- shows the unaffected premolars and second molars. OPG: Orthopantomogram.

On intra‑oral examination, extensive atypical post‑eruptive breakdown (PEB) of enamel was seen in all four FPMs and maxillary incisors, affecting 80–85% of the surfaces. Demarcated opacities had distinct borders with the adjacent sound enamel. Mandibular permanent incisors were fragile with an irregular pitted enamel surface, and the erupting mandibular canine showed PEB on the incisal edge. All the erupting first premolars were sound and unaffected with multiple severely carious deciduous molars (Figures 1 and 2). On radiographic examination, Orthopantomogram (OPG) revealed radiolucency on the affected teeth with ‘moth-eaten appearance of the crown’. No pulpal or periradicular involvement was noted in any of the FPMs and incisors. Both unerupted maxillary canines showed radiolucency less than enamel in the incisal region with irregular enamel margins showing hypomineralisation. All the premolars and second molars appeared normal radiographically (Figure 3). The patient had poor oral hygiene with moderate plaque and calculus.

Based on the patient’s chief complaint, medical and dental history, along with the clinical examination and radiographic findings, the patient presented with a mixed enamel defect pattern. Teeth 16, 26, 36, 46, 11, 21, 12, and 22 demonstrated characteristic features of MIH, with qualitative enamel defects. In contrast, teeth 31, 32, 33, 41, 42, and 43 exhibited signs more consistent with enamel hypoplasia, evidenced by reduced enamel thickness and altered morphology, indicating a quantitative defect. This combined presentation suggests a complex developmental enamel disturbance, with both hypomineralisation affecting FPMs and maxillary incisors and hypoplasia affecting the mandibular anterior dentition. Severity of MIH was assessed using the European Academy of Paediatric Dentistry (EAPD) diagnostic criteria and severity index [15]. Treatment needs were determined based on the Würzburg concept using the MIH Treatment Need Index (MIH-TNI) [26]. Teeth 16, 26, 36, 46, 11, 21, 12, and 22 were classified as severe and assigned MIH-TNI category 4b-D, indicating a need for full-coverage restorative intervention. Due to the structural weakness and compromised enamel quantity in 31, 32, 41, and 42, a full-coverage restorative approach was adopted to protect the remaining enamel and prevent further breakdown.

The patient’s mother was informed about the diagnosis, followed by counselling, and was motivated for proper treatment and longer follow‑ups. In the first visit, motivation was given to improve oral hygiene and modify the child’s dietary habit followed by oral prophylaxis and professional topical application of Duraphat^®^ (Colgate Oral Pharmaceuticals, New York, NY, USA) fluoride varnish. The patient was advised to use the remineralising agent casein phosphopeptide amorphous calcium phosphate (CPP‑ACP) (GC tooth mousse, GC, Tokyo, Japan) for self‑application at home on the affected teeth. An initial treatment plan was formulated, consisting of direct composite resin restorations for the anterior teeth and stainless steel crowns (SSC) for the affected posterior teeth. However, the patient exhibited reluctance regarding the frequent fracture and aesthetics of the anterior composite resin restorations and the metallic appearance of the SSCs. Given the patient’s aesthetic concerns and preference for a more natural appearance, the treatment plan was subsequently modified. CAD/CAM-fabricated PMMA crowns were proposed as an aesthetic restorative solution, and the ‘No prep crown technique’ was chosen in order to prevent further removal of already compromised enamel and exposing more dentinal surface.

In the second visit, polyvinyl siloxane (PVS; Express™ VPS Impression Material, 3M™ ESPE, St. Paul, MN, USA) impressions were taken of both dental arches to accurately record anatomical and occlusal relationships. The impressions were sent to the laboratory, where stone casts were fabricated and subsequently scanned using the Dentsply Sirona inEos X5 optical scanner to generate digital models. Full coronal restorations were then designed using Exocad GmbH CAD software, ensuring appropriate proximal contacts, occlusion, and marginal fit. The crowns were designed with a uniform thickness of approximately 1–1.5 mm to provide optimal strength and aesthetics, and all four anterior crowns were fabricated as a single unit. This design was selected to improve biomechanical strength and load distribution, enhance stability, and ensure superior aesthetic continuity across the anterior segment. The final designs were milled using the Dentsply Sirona inLab MC X5 milling unit from Dentura PMMA A1 shade blocks. After milling, the crowns were finished and polished, followed by intraoral try-in for assessment of fit and occlusion. The definitive cementation was performed using 3M™ RelyX™ U200 Automix Self-Adhesive Dual-Cure Resin Cement, following the manufacturer’s recommended protocol. The patient was advised to attend a check-up 1 week later, followed by every 3–6 months for professional topical fluoride application on canines.

In the follow‑up visits, the patient reported complete relief from dentinal hypersensitivity as well as bad breath. The patient’s mother reported that the treatment had a positive psychological impact on her son, with improvement in self-confidence and overall demeanour contributing to improved social interactions and a more confident attitude in school and peer environments.

All the PMMA crowns were intact and functional (Figure 3). Both patient and parent were satisfied at the end of 24 months. The patient has been kept on long term follow-up until the complete eruption of all teeth for management of canines and orthodontic treatment in both dental arches. Further, follow-up will continue until definitive treatment planning and replacement with permanent crowns.

## Discussion

Ameloblasts are extremely sensitive during the amelogenesis process, thus any physiological or environmental disruption that takes place during this mineralisation or maturation stage—that is, during gestation and infancy—causes a qualitative deficiency of enamel in the FPMs and incisors [4, 15, 27]. The chronology or time period of the disruption or injury determines the type of dentition and teeth affected. In addition to FPMs and incisors, canines were also impacted in our study. Interestingly, similar findings were reported by Fagrell et al. [28] and Kevrekidou et al., [29] reinforcing evidence that MIH does not exclusively involve incisors and FPMs; however, MIH does not require symmetrical or homologous involvement as seen in fluorosis. The defect pattern depends on the timing and severity of the developmental insult rather than uniform systemic exposure.

The exact pathogenesis of MIH is currently unknown; prenatal, perinatal, postnatal, genetic and environmental factors are among the theories put up in the literature [4]. However, in our study, the most plausible reason for the MIH was postnatal severe hypocalcaemia secondary to vitamin-D deficiency in his late first year of life. This is consistent with literature suggesting that postnatal metabolic disturbances may play a critical role in MIH development [30].

Management of MIH-affected teeth remains complex due to inherent challenges such as dentinal hypersensitivity, reduced anaesthetic efficacy, frequent marginal breakdown, and difficulty achieving durable bonding to hypomineralised enamel [31]. These factors necessitated an individualised treatment strategy focused initially on symptom control and stabilisation rather than immediate full rehabilitation [32]. Although the patient presented at a young age with extensive damage, the early phase of care emphasised conservative measures, including maintaining oral hygiene, dietary modification, and routine clinical monitoring to minimise plaque accumulation and prevent further deterioration.

Preventive therapy was performed by current evidence supporting the use of fluoride varnish and CPP-ACP in MIH. Topical fluorides act by forming a temporary protective layer and enhancing remineralisation, while CPP-ACP maintains a bioavailable calcium-phosphate environment at the enamel surface, offering synergistic benefits when combined [15, 33, 34]. Biondi et al. [35] demonstrated differential outcomes among varnish formulations, with Duraphat^®^ proving most effective in moderate lesions and Clinpro^®^ performing better in mild cases. Similarly, Olgen et al. [36] reported that all tested remineralisation agents improved lesion status over a 24-month period, with calcium- and phosphate-containing pastes offering the most sustained improvement in yellow-brown defects. Based on this evidence, fluoride varnish (Duraphat) was applied prior to crown placement to strengthen enamel and reduce sensitivity, and the patient was additionally instructed to use CPP-ACP for ongoing remineralisation of non-restored affected teeth.

Several restorative options have been described for MIH, including stainless steel crowns, conventional and resin-modified glass ionomer cement, composite resin, and ceramic crowns [37]. In this case report, PMMA crowns were chosen for both anterior and posterior rehabilitation instead of stainless-steel crowns or composite restorations primarily due to aesthetic considerations, as the patient and parent preferred tooth-coloured restorations over a metallic appearance. Composite restorations were also deemed unsuitable due to the extensive structural loss and the presence of hypomineralised enamel, which compromises adhesion and increases the risk of restoration failure. Additionally, improving the retention of composite restorations in MIH-affected teeth often requires removal of the compromised enamel; however, considering the patient’s limited compliance and the extent of enamel breakdown, a full-coverage restoration was considered more predictable and also prevents further structural loss and provides long-term functional stability [37].

CAD/CAM-fabricated PMMA crowns offer efficient fabrication along with good colour stability, precision, and fit, resulting in excellent marginal integrity. These crowns provide reliable shade matching and acceptable aesthetic outcome, particularly in the anterior and posterior teeth that are visible during function [38, 39]. These crowns also promote a positive fibroblast response, which results in a good soft-tissue seal, minimal bacterial adhesion, and a prolonged presence of the material in the oral cavity [40]. Also, they are suitable for proper function, aesthetic integration, and minimising the risk of complications like secondary caries or crown dislodgement [40]. However, a potential drawback of PMMA crowns is their lower long-term durability, particularly under high occlusal load [41]. Stainless-steel crowns remain the standard for severely affected posterior MIH cases due to their proven longevity [37], but in this case, the balance between functional needs, aesthetics, and patient preference justified the use of PMMA crowns.

The revised plan was well-received by the patient and parent, and the restorations were successfully placed. The patient expressed satisfaction with both the functional and aesthetic outcomes of the treatment. The mother’s observations on the increased self-confidence of her son suggest that, beyond functional rehabilitation, addressing the aesthetic concerns through minimally invasive techniques can significantly improve the psychological well-being, self-esteem, and social confidence in adolescent MIH affected patients.

Further research with larger sample sizes and standardised outcome measures is necessary to validate these findings and create evidence-based guidelines for aesthetic rehabilitation in paediatric patients with MIH, especially considering the excellent outcome obtained in this case report with a 24-month follow-up. This case report’s limitation includes a lack of long-term follow-up to assess the survival rate of MIH teeth and PMMA crown retention in paediatric patients treated for a longer period, which is an area of future research.

## Conclusion

This case report underscores the importance of early diagnosis and individualised management of MIH in children, particularly when aesthetic concerns and psychological well-being are affected. A patient-centred and conservative therapeutic approach may provide both clinical and emotional benefits in addressing developmental enamel defects. The use of minimally invasive, CAD/CAM-fabricated PMMA crowns may serve as a functional and aesthetically acceptable treatment option in selected cases; however, the long-term performance of PMMA restorations in MIH-affected dentition remains uncertain. Further studies with larger sample sizes and extended follow-up periods are needed to clarify their durability and define their role within comprehensive MIH management protocols.

## Data Availability

Not applicable.
